# Vaccination hesitation in children under five years of age: a scoping review

**DOI:** 10.1590/0034-7167-2022-0707

**Published:** 2023-11-27

**Authors:** Eugênio Barbosa de Melo, Priscilla Dantas Almeida, Beatriz Mourão Pereira, Paulo de Tarso Moura Borges, Elucir Gir, Telma Maria Evangelista de Araújo

**Affiliations:** I Universidade Federal do Piauí. Teresina, Piauí, Brazil; II Universidade Federal do Amazonas. Manaus, Amazonas, Brazil; III Universidade de São Paulo. Ribeirão Preto, São Paulo, Brazil

**Keywords:** Vaccine Hesitation, Vaccination Coverage, Vaccination Refusal, Parents, Children, Vacilación a la Vacunación, Cobertura de Vacunación, Negativa a la Vacunación, Padres, Niños, Hesitação Vacinal, Cobertura Vacinal, Recusa de Vacinação, Pais, Crianças.

## Abstract

**Objectives:**

to synthesize scientific evidence on vaccine hesitancy in children under five years of age and its associated factors.

**Methods:**

a scoping review, conducted according to the methodological structure proposed by the JBI. Searches were carried out in the Latin American and Caribbean Center on Health Sciences Information, Scientific Electronic Library Online and PubMed databases, including gray literature. Studies in English, Spanish and Portuguese were included, without temporal delimitation. Editorials, studies that did not address vaccine hesitancy in children under five years of age and were not aligned with the objective and research question were excluded. The sample consisted of 18 articles.

**Results:**

misinformation, concern about adverse effects, distrust about efficacy, affliction regarding administration simultaneously, and insecurity in relation to the laboratories were the reported reasons.

**Conclusions:**

strategies are needed to combat the lack of information about immunobiological agents, as misinformation was the main factor in parents’ vaccine hesitation.

## RESUMO

**Objetivos:**

sintetizar evidências científicas sobre hesitação vacinal em crianças menores de cinco anos e seus fatores associados.

**Métodos:**

revisão de escopo, conduzida conforme a estrutura metodológica proposta pelo JBI. Realizaram-se buscas nas bases Centro Latino-Americano e do Caribe de Informação em Ciências da Saúde, *Scientific Electronic Library Online* e PubMed, incluindo literatura cinzenta. Foram incluídos estudos em inglês, espanhol e português, sem delimitação, temporal. Foram excluídos editoriais, estudos que não abordaram hesitação vacinal em menores de cinco anos e não estiveram alinhadas com o objetivo e questão de pesquisa. A amostra foi composta por 18 artigos.

**Resultados:**

desinformação, preocupação com efeitos adversos, desconfiança sobre eficácia, aflição quanto à administração simultaneamente e insegurança em relação aos laboratórios foram os motivos relatados.

**Conclusões:**

são necessárias estratégias de combate à carência de informações acerca dos imunobiológicos, pois a desinformação foi o fator principal na hesitação vacinal dos pais.

## RESUMEN

**Objetivos:**

sintetizar la evidencia científica sobre la reticencia vacunal en menores de cinco años y sus factores asociados.

**Métodos:**

revisión de alcance, realizada según la estructura metodológica propuesta por el JBI. Las búsquedas se realizaron en el Centro Latinoamericano y del Caribe de Información en Ciencias de la Salud, *Scientific Electronic Library Online* y PubMed, incluida la literatura gris. Se incluyeron estudios en inglés, español y portugués, sin delimitación temporal. Se excluyeron editoriales, estudios que no abordaran la reticencia vacunal en menores de cinco años y que no estuvieran alineados con el objetivo y pregunta de investigación. La muestra estuvo compuesta por 18 artículos.

**Resultados:**

la desinformación, la preocupación por los efectos adversos, la desconfianza en la eficacia, la angustia por la administración simultánea y la inseguridad por los laboratorios fueron los motivos informados.

**Conclusiones:**

se necesitan estrategias para combatir la falta de información sobre inmunobiológicos, ya que la desinformación fue el principal factor en la duda vacunal de los padres.

## INTRODUCTION

Among the health promotion and protection strategies, disease prevention is one of the fundamental pillars of the Brazilian public health system, since preventing diseases from taking hold increases individuals’ and populations’ quality and life expectancy. Moreover, it generates considerable savings in terms of cost in the country’s public health, as it avoids expenses related to parents’ diagnosis, treatment and rehabilitation.

Thus, immunizing individuals through vaccination represents one of the most cost-effective primary prevention measures with a clear positive impact on collective health, reducing morbidity and mortality from infectious diseases, especially in children up to one year of age^(1)^.

The success of the Brazilian National Immunization Program (PNI - *Programa Nacional de Imunizações*), whose performance and scope are compared to those of developed countries, took place through vaccination actions that resulted in a decline in morbidity and mortality from communicable diseases in the country, the eradication of poliomyelitis in 1989 and rubella and measles elimination certifications, received in 2015 and 2016, respectively. However, the high vaccination coverage (VC) rates achieved by the PNI have fallen in recent years, resulting in the resurgence of vaccine-preventable diseases, such as measles^(2-5)^.

The decline in the national VC was relevant between 2015 and 2019, since there was a significant drop in the percentages of VC: polio, MMR (first dose), BCG, pentavalent, hepatitis B (in children up to 30 days), hepatitis A, pneumococcal, meningococcal C and human rotavirus. In this context, 10,330 cases of measles were registered in 2018 and 20,901 in 2019, in different regions of the country, causing Brazil to lose, in 2019, the certificate of disease eradication. It is important to highlight that the COVID-19 pandemic contributed to the drop in VC during 2020^(5-6)^.

In addition to this, it is noteworthy that the decline in VC may be related, among other things, to the geographic location of health units in relation to users’ residence, social determinants, discontinuity of immunobiological agent supply, opening hours of vaccine rooms, and, above all, vaccine hesitancy (VH), defined as the delay in accepting or refusing recommended vaccines, despite their availability in health services^(4)^. Such behavior has been influenced by cultural, social, religious and economic issues as well as by the lack of information and/or misinformation about aspects involving the immunization processes^(4,7-10)^.

In Brazil, although VH is a recognized and increasingly evident problem, few studies have been developed about VH and it is still not possible to effectively identify which factors are associated with individuals with this type of behavior^(11-13)^. Thus, this study helps to identify and understand the factors that corroborate VH among fathers, mothers or guardians of children under five years of age, providing indispensable information for the planning of nursing actions, especially those developed in Primary Health Care, which strengthen VC in the child population.

## OBJECTIVES

To synthesize available scientific evidence on VH in children under five years of age and its associated factors.

## METHODS

### Ethical aspects

Since it is research that used public domain data, it was not necessary to submit the review for appreciation by the Research Ethics Committee (REC).

### Study design, period and place

This is a scoping review whose objective is to synthesize the findings of scientific research, about a certain research problem, without analyzing the methodological quality of included studies. It was developed based on the methodological framework proposed by the JBI^(14)^, following the Preferred Reporting Items for Systematic reviews and Meta-Analyses extension for Scoping Reviews (PRISMA-ScR) Checklist^(15)^. This review had its protocol registered on the Open Science Framework (OSF) platform and can be accessed at https://osf.io/nzyqp/.

As proposed by the JBI, the following steps were followed: define and align the objective and questions; develop and align inclusion criteria with objective and questions; describe the planned approach to evidence search, selection, data extraction and evidence presentation; search, select, extract and analyze evidence; present the results; summarize the evidence against the purpose of the review by drawing conclusions; and observe the implications of the findings^(14)^.

To elaborate the research question, the mnemonic PCC (Participant, Concept and Context) was used. Participants are fathers, mothers or guardians of children under five years of age; concept is VH including associated factors; and context is childhood vaccination. In this regard, the question of this scoping review was: what is the available evidence on vaccine hesitancy in children under five years of age and its associated factors?

The bibliographic survey was carried out between May and June 2022, in the following databases and bibliographic index: Latin American and Caribbean Center on Health Sciences Information (BIREME); Scientific Electronic Library Online (SciELO); and PubMed. As for gray literature, it was searched in the Coordination for the Improvement of Higher Education Personnel (CAPES - *Coordenação de Aperfeiçoamento de Pessoal de Nível Superior*) Theses and Dissertations Catalog and Google Scholar. The search strategy in the databases was conducted from Health Sciences Descriptors (DeCS)/Medical Subject Headings (MeSH), which were combined using Boolean operators AND and OR. In each database, three separate searches were performed, as described in Chart 1.

**Chart 1:** Search syntax of articles in data sources, Teresina, Piaui, Brazil, 2022

Sources	Syntax
BIREME SciELO PubMed CAPES Theses and Dissertations Catalog Google Scholar	*Hesitação Vacinal* OR Vaccination Hesitancy
	*Hesitação Vacinal* AND *Fatores Associados*
	Vaccination Hesitancy AND Associated factors

Cross-sectional studies, randomized clinical trials and systematic reviews, published online in full, without time restriction, in Portuguese, English and Spanish were included. Editorials, publications that did not address VH in children under five years of age, and publications that were not aligned with the objective or addressed the research question were excluded.

The search was carried out by two independent reviewers, simultaneously, who carried out the initial screening, based on the screening of titles and, later, reading the abstracts using the eligibility criteria as a guide, and all identified references were imported into a reference management software, Zotero 6^(16)^, for selecting, organizing and archiving articles. All duplicate articles were counted only once. Differences regarding the inclusion of a study were discussed and resolved between the reviewers. Ties were resolved by a third reviewer who, after reading the entire material, broke the tie to compose the final sample.

Data were collected using a data mapping form, created in Microsoft Word and based on the manual provided by JBI^(17)^. The form was initially tested by two independent reviewers using a random sample of 10% of included studies to ensure consistency and accuracy. Data extraction from eligible documents was performed independently by two reviewers. Data analysis was conducted to identify themes and subthemes, which were grouped, summarized and presented as a narrative.

The classification of the scientific evidence of the articles that composed the sample was based on the levels of evidence established by the JBI^(18)^.

## RESULTS

**Figure 1:**
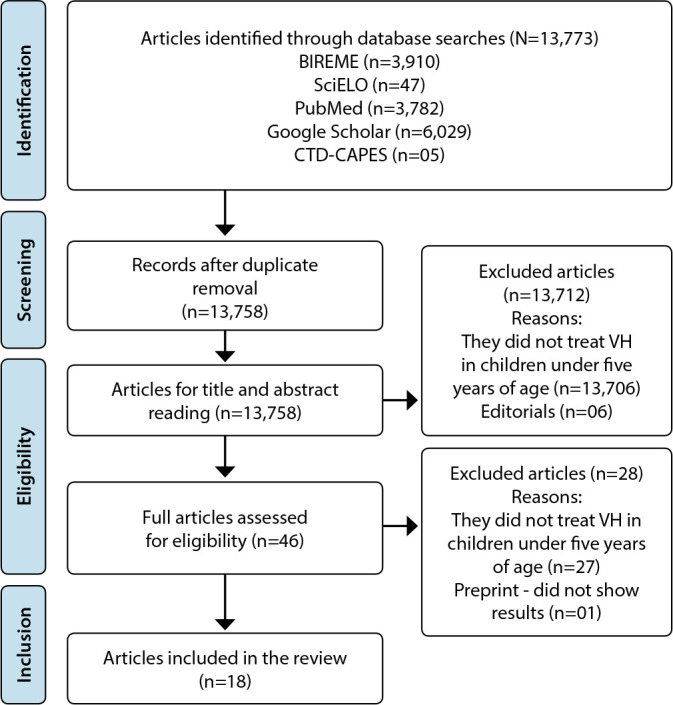
PRISMA flow diagram, Teresina, Piaui, Brazil, 2022

The entire study selection process was mapped according to the PRISMA^(19)^ flowchart (Figure 1). Initially, 13,773 publications were identified, distributed in the BIREME (n=3,910), SciELO (n= 47), PubMed (n=3,782), Google Scholar (n=6,029) and CTD-CAPES (n=05) databases, excluding those who presented repeated (n=15). Then, editorials (n=06) were excluded as well as studies that did not address VH in children under five years of age, by reading the titles (n=12,722) and abstracts (n=984), 46 selected articles remaining. Of these, 27 were excluded, as they did not treat VH in children under five years of age and one preprint, since it did not show results. In the end, the sample consisted of 18 articles.

The sample consisted of articles published in 2017 (03), 2018 (02), 2019 (05), 2020 (02), 2021 (03) and 2022 (03). There was no prevalence regarding the country in which the studies were carried out, since the sample was uniformly distributed among 13 countries. Regarding the study design, cross-sectional studies prevailed (14), classified at level 4.b of evidence (Chart 2).

**Chart 2:** Characterization of studies included in the review, Teresina, Piaui, Brazil, 2022

Id	Title	Year	Country	Design	LoE^(18)^
S1^(20)^	Vaccine hesitancy among parents in a multi-ethnic country, Malaysia	2017	Malaysia	Cross-sectional study	4.b
S2^(21)^	Longitudinal Trends in Vaccine Hesitancy in a Cohort of Mothers Surveyed in Washington State, 2013-2015	2017	USA	Randomized clinical trial	1.c
S3^(22)^	Childhood vaccine refusal and hesitancy intentions in Croatia: insights from a population-based study	2017	Croatia	Cross-sectional study	4.b
S4^(23)^	Vaccine confidence and hesitancy in Brazil	2018	Brazil	Cross-sectional study	4.b
S5^(24)^	Measuring childhood vaccination acceptance of mother in Zhejiang province, East China	2018	China	Cross-sectional study	4.b
S6^(25)^	Vaccine hesitancy among Saudi parents and its determinants	2019	Saudi Arabia	Cross-sectional study	4.b
S7^(26)^	Parent perspectives on childhood vaccination: How to deal with vaccine hesitancy and refusal?	2019	Italy	Cross-sectional study	4.b
S8^(27)^	Why do some Korean parents hesitate to vaccinate their children?	2019	Korea	Cross-sectional study	4.b
S9^(28)^	A meta-synthesis study of the key elements involved in childhood vaccine hesitancy	2019	Italy	Systematic review	1.b
S10^(29)^	Exploring parents’ reasons for incomplete childhood immunisation in Indonesia	2019	Indonesia	Cross-sectional study	4.b
S11^(30)^	Factors affecting vaccine hesitancy among families with children 2 years old and younger in two urban communities in Manila, Philippines	2020	Philippines	Cross-sectional study	4.b
S12^(31)^	Addressing Parental Vaccine Hesitancy towards Childhood Vaccines in the United States: A Systematic Literature Review of Communication Interventions and Strategies	2020	USA	Systematic review	1.b
S13^(32)^	Prevalence and Determinants of Vaccine Hesitancy in Aseer Region, Saudi Arabia	2021	Saudi Arabia	Cross-sectional study	4.b
S14^(33)^	Vaccine Hesitancy: Characteristics of the Refusal of Childhood Vaccination in a Peruvian Population	2021	Peru	Cross-sectional study	4.b
S15^(34)^	Vaccine hesitancy among mothers of under-five children in Coastal South India: a facility-based cross-sectional study [version 3; peer review: 2 approved]	2021	India	Cross-sectional study	4.b
S16^(35)^	Vaccine Hesitancy for Childhood Vaccinations in Slum Areas of Siliguri, India	2022	India	Cross-sectional study	4.b
S17^(36)^	I’m a mother, therefore I question”: Parents’ legitimation sources of and hesitancy towards early childhood vaccination	2021	Turkey	Cross-sectional study	4.b
S18^(37)^	Opinions of parents concerning childhood vaccine refusal	2021	Turkey	Cross-sectional study	4.b

*Id - identification; S - study; LoE - level of evidence.*

Cross-sectional studies were intended, in summary, to investigate the prevalence and determinants of VH in parents of children under five years of age. With the exception of the reviews (S9 and S12), the survey samples were made up of children’s parents or guardians (Chart 3).

**Chart 3:** Description of studies according to objective, population and sample, Teresina, Piaui, Brazil, 2022

Id	Objective(s)	Population	Sample
S1^(20)^	Assess the Parent Attitudes about Childhood Vaccines (PACV) questionnaire test-retest reliability in Malay and determine the prevalence of VH among parents and its associations with sociodemographic characteristics.	Parents^*^ aged 18 years or older, with at least one child under the age of seven, or pregnant women who visited the pediatric and/or antenatal clinics during data collection	545 participants
S2^(21)^	Assess trends in parental VH during the first two years of their children’s lives in a cohort of mothers in Washington state.	Mothers of children up to two years old	237 mothers
S3^(22)^	Estimate the prevalence and sociodemographic and sociocultural determinants of refusal intentions and childhood VH among Croatian adults.	Individuals aged between 18 and 88 years	1,000 participants
S4^(23)^	Assess confidence and VH in Brazil as part of a broader project to map vaccine confidence worldwide.	Graduated dental surgeons, parents^*^/companions of children who attended oral health outpatient clinics during data collection	952 participants
S5^(24)^	Assess VH among mothers and examine risk factors associated with maternal intention to vaccinate in Zhejiang Province.	Mothers of children aged 24 to 35 months	770 mothers
S6^(25)^	Assess the prevalence of VH and its determinants among Saudi parents.	Parents^*^ who attended an outpatient pediatric clinic during the data collection period	500 participants
S7^(26)^	Assess attitudes about childhood vaccines and vaccine refusal or delay among parents and assess the role played by variables mapped as potential determinants.	Parents^*^ of students aged up to five who attended kindergartens during the data collection period	575 participants
S8^(27)^	Study the reasons why some Korean parents hesitate to vaccinate their children, applying the health belief model.	Parents^*^ of children attending alternative education preschools and primary schools during the data collection period	141 participants
S9^(28)^	Summarize the evidence on VH in childhood from the perspective of parents.	Qualitative articles focused on vaccine refusal and VH	27 articles
S10^(29)^	Explore the underlying factors behind why Indonesian parents choose not to immunize or partially immunize their children.	Parents^*^ or guardians of partially and intentionally unvaccinated children	16 participants
S11^(30)^	Determine factors influencing VH among parents and caregivers of children aged two years and younger in selected urban communities in Manila.	Parents^*^ and caregivers of children up to two years of age	110 participants
S12^(31)^	Develop a catalog of interventions and health communication strategies to address and prevent parental VH.	Articles published from January 2008 to October 2019	75 articles
S13^(32)^	Assess the general population’s knowledge of vaccines to determine the prevalence of VH in the Aseer region, southern Saudi Arabia.	Parents^*^ from the Aseer region, Saudi Arabia	796 participants
S14^(33)^	Investigate the characteristics of the vaccination decision-making process that may result in the refusal of childhood immunization in parents from Lima, Peru.	Parents^*^ or guardians of children treated at a private pediatric practice	552 participants
S15^(34)^	Identify the prevalence and reasons for VH among mothers of children under five in Mangalore.	Mothers of children under five	172 participants
S16^(35)^	Discover the proportion and factors that contribute to VH, in childhood vaccination, in slums of the city of Siliguri.	Parents^*^ of children under five years of age	194 participants
S17^(36)^	Understand how mothers decide about childhood immunization and the attitudes, perceptions and beliefs underlying these decisions.	Mothers of children under five years of age	23 participants
S18^(37)^	Reveal the opinions, knowledge and attitudes of parents who refuse childhood vaccination.	Parents^*^ of children under four years of age	590 participants

Id - identification; S - study;

* fathers and mothers.

The analysis of the most relevant results found in the studies allowed identifying the main determinants of VH. These were synthesized and distributed into four categories: (1) Misinformation and fake news; (2) Adverse effects; (3) Vaccine efficacy and safety; and (4) Religious Beliefs. The distribution of studies, according to the content presented in the results, and their respective categories, is described in Chart 4.

**Chart 4:** Categorization of results found in studies, Teresina, Piaui, Brazil, 2022

Order	Category of results	Articles	Total
1	Misinformation and fake news	S1, S2, S5, S6, S7, S8, S9, S10, S11, S12, S14, S15, S16, S17 and S18	15 studies
2	Adverse effects	S1, S2, S4, S7, S9, S10, S11, S12, S13, S14 and S15.	11 studies
3	Vaccine safety and efficacy	S1, S2, S4, S6, S7, S9, S10, S11, S12, S14, S17 and S18.	12 studies
4	Religious beliefs	S3, S9, S10, S17 and S18.	05 studies

*S - study.*

### Misinformation and fake news

This category was present in 15 studies (S1, S2, S5, S6, S7, S8, S9, S10, S11, S12, S14, S15, S16, S17 and S18). It was noticed that hesitant parents had characteristics alluding to lack of information and/or misinformation (here understood as having divergent information about immunobiological agents). Many have demonstrated belief in conspiracy theories; concern with the number of vaccines intended for the child population; belief that illnesses are not so dangerous; general questions about the vaccination schedule and vaccines; exposure to negative media information; lack of clarity in the information provided by the government, health professionals and health institutions; and doubts regarding sources of reliable information on immunobiological agents.

### Adverse effects

Concern about possible serious adverse effects was present in 11 studies (S1, S2, S4, S7, S9, S10, S11, S12, S13, S14 and S15). Studies revealed that hesitant parents/guardians reported having high concern related to reactions such as fever, allergies, disabilities, autism, paralysis, seizures and even death.

### Vaccine safety and efficacy

In 12 of the analyzed studies (S1, S2, S4, S6, S7, S9, S10, S11, S12, S14, S17 and S18) concern/distrust about vaccine efficacy and safety was mentioned by participants. Some of them showed concern about the safety of administering vaccines to children. Furthermore, the application of two or more vaccines, administered at the same time in different anatomical regions and routes of administration, is another factor that causes extreme distress among parents. There are also those who said they did not believe in the effectiveness of vaccines and also showed insecurity in relation to the laboratories that manufacture immunizers.

### Religious beliefs

In five studies (S3, S9, S10, S17 and S18), evidence was found about the religious influence on the behavior of hesitant parents. Children of Islamic parents were not vaccinated due to the belief that pork-derived products are part of the composition of immunobiological agents. In other cases, some parents stated that exposing children to diseases would build immunity, since diseases are natural and that, therefore, “God would decide whether the child would be sick or not”. Added to this, there is the fact that some parents reported believing more in alternative medicine than in immunization through vaccines.

## DISCUSSION

Although this review carried out a synthesis and categorization of the results related to the causes of VH, the reasons that lead parents to postpone or refuse their children’s vaccination are multiple and complex. However, the reasons have been presented in a dissociated way in the present work, it is essential to remember that, when it comes to hesitant parents, the reasons that determine VH can also be correlated.

The lack of pertinent and adequate information causes people to seek help in the media, especially on the internet. This behavior triggers a series of disorders due to the flood of available information, whether true or not. Although social media are undisputed means of mass communication, they have also been a huge source of untrue information and, consequently, harmful to public health^(38)^.

Fake news about vaccines gained greater notoriety during the advent of the COVID-19 pandemic. With social isolation required by health authorities, people have become hyperconnected to social media most of the time, virtual environments in which most people still do not know how to differentiate between false and true news. Therefore, the problem revolves around the questionable quality of the contents accessed^(26,39-40)^.

The difficulty in dealing with the ambiguity of available information was reported in a systematic review and meta-synthesis, with articles published until 2018. The study revealed that hesitant parents stated that there was no objective information about vaccines, since the available information is often unreliable, due to the fact that it is fragmented, contradictory and biased^(41)^.

Health professionals play an important role in the fight against VH, as long as they adopt postures and speeches that aim to positively influence the population’s behavior. It is recognized that its recommendations, when shared clearly and in language accessible to the most varied social classes, awakens confidence in vaccines in users^(42)^.

In this same context of mistrust, part of the parents justifies VH based on mistaken reports that cause concern about the possible serious adverse effects caused by immunobiological agent administration. It is important to highlight that, similarly to any treatment, vaccines can trigger serious complications, such as severe allergic reactions, even though they are not frequent. However, some of the adverse effects such as autism, infertility and disabilities associated with the immunization process have already been refuted by science^(32,43-44)^.

Hesitant parents harbor concerns about vaccine safety, as surveys show that 40-52% of parents had concerns about vaccinating their children^(45-46)^. The belief in the ineffectiveness or safety of vaccines is also associated with the belief that immunobiological agents are nothing more than a product that pharmaceutical industries use exclusively to profit^(26)^. This association can make people believe that vaccines are not made up of compounds capable of stimulating the immune system, since, according to the belief, they would be a type of placebo.

Allied to this, there is the fact that many individuals agree that the number of vaccines administered to children is very high. Although the number of vaccines given to children has increased significantly over the past decade, scientific evidence clearly states that there is no upper limit to the number of vaccines that can be given simultaneously^(33)^.

According to the report “Adverse events Associated with childhood vaccines”, one of the Institute of Medicine of the United States, children are exposed to many foreign antigens every day when eating food. Furthermore, numerous bacteria live in the mouth and nose, exposing the immune system to even more antigens. Given these normal events, it seems unlikely that the number of antigens contained in childhood vaccines pose a risk to the immune system^(47)^.

Religiosity and beliefs in alternative therapies that lead to VH are a major obstacle for health authorities that seek to increase childhood vaccination rates, given the difficulty of reversing such opinions/behaviors^(48)^. A study of Islamic parents revealed that children were not given any vaccines as their parents believed they contained pork products, and as Muslims are prohibited from eating pork, children were not vaccinated, because vaccines would be able to destroy the healthy cells of their children, due to the presence of porcine substance^(29)^.

Allied to religious issues is the fact that some hesitant parents believe that vaccines represent an ‘unnatural’ approach to health, interfering with the body’s ability to naturally protect itself against disease, using the example that in times when vaccines did not exist, people were naturally healthy^(29,41)^. It would be interesting for these parents to remember that diseases such as smallpox, polio and Spanish flu existed in the past and that they were only eradicated after the advent of vaccination.

### Study limitations

As a limitation, mention is made of the lack of national studies on the subject, which impairs the comparison with other realities as well as hinders a more accurate visualization of VH in the current Brazilian scenario. In this context, it is pertinent to carry out new studies at the national level. Moreover, it is important to highlight the scarcity of studies with a high level of evidence in the sample, since there was a prevalence of cross-sectional studies.

### Contributions to nursing

The results, in addition to adding knowledge to the subject, provide important information about the causes of VH, to those health professionals who work with vaccination in general. In this regard, nursing professionals, specifically primary health care nurses, for being directly and permanently involved with the immunization process, will find subsidies to guide the creation of strategies aimed at minimizing VH, such as permanent health education, production of applications for smartphones, folders, booklets, etc., with a view to clarifying/informing the population about immunobiological agents, consolidating the relevance of nursing in maintaining public health quality.

## CONCLUSIONS

Hesitant parents’ behavior regarding VH is determined by a complex of factors. This review synthesized the most relevant results of analyzed studies, making clear the need to develop tools with the potential to combat, in particular, the lack of information about immunobiological agents, since misinformation, characterized as the absence or misunderstanding of available information, concerns related to adverse reactions and safety in vaccine administration, and religious influence, were identified as the main factors in the VH of parents of children under five years of age. In an era marked by easy access to information and access to the means of communication, it is extremely important to create tools that provide accurate, logical, organized information in accessible language to the population.

## Data Availability

https://doi.org/10.48331/scielodata.GV2AAB
